# Influence of pension availability on the association between work conditions and labor market exit for health reasons: evidence from a Taiwanese older adults cohort

**DOI:** 10.1186/s12889-025-22215-3

**Published:** 2025-03-17

**Authors:** Hung-Yi Chiang, Yawen Cheng, Hans Martin Hasselhorn, Chih-Cheng Hsu, Yun-Chieh Yang, Wan-Ju Cheng

**Affiliations:** 1 Department of Emergency Medicine, Jen Ai Hospital Dali Branch, 483 Dong Rong Road, Dali, Taichung, Taiwan; 2 Institute of Health Policy and Management, College of Public Health, National Taiwan University, 17 Xu-Zhou Road, Taipei, Taiwan; 3 Department of Occupational Health Science, University of Wuppertal, Gaußstraße 20, Wuppertal, 42119 Germany; 4 Institute of Population Health Sciences, National Health Research Institutes, 35 Keyan Road, Miaoli, Taiwan; 5 National Center for Geriatrics and Welfare Research, National Health Research Institutes, 35 Keyan Road, Miaoli, Taiwan; 6 Department of Psychiatry, China Medical University Hospital, 2 Yude Road, Taichung, Taiwan; 7 Department of Public Health, China Medical University, Jingmao Rd, 100 Sec.1, Taichung, Taiwan

**Keywords:** Pension, Population aging, Psychosocial work conditions, Retirement

## Abstract

**Background:**

While the impact of poor working conditions on workers' intention to leave the job is well-established, limited research has examined how the availability of pension benefits influences the association between adverse psychosocial work conditions and labor market exit for health reasons among middle-aged and older workers. This study explored the association of psychosocial and physical work conditions with labor market exit for health reasons among individuals with varying pension statuses.

**Methods:**

This study utilized data from the Healthy Aging Longitudinal Study in Taiwan (HALST), which investigated reasons for labor market exit among 2,143 adults aged 55 and older. Work conditions were aggregated by occupation based on data from the Occupational Safety and Health Surveys, which included nationally representative employees, and subsequently linked to HALST data. We examined the differential impact of psychosocial and physical work conditions on health-related labor market exit, compared to old age retirement, among individuals with and without pension coverage.

**Results:**

Among 2,143 study participants, 7.3% left the labor market due to health reasons, and 39.9% reported not having a pension. Individuals with low job control (adjusted odds ratio [aOR] = 2.23, 95% confidence interval [CI] = 1.05 to 4.73) and high physical demands (aOR = 2.72, 95% CI = 1.26 to 5.85) were more likely to exit the labor market for health reasons compared to old age retirement. Among participants without a pension, adverse work conditions were significantly associated with labor market exit for health reasons.

**Conclusions:**

Adverse work conditions were associated with labor market exit for health reasons particularly among older adults without pension coverage. Implementing policies to improve psychosocial work conditions and enhance the pension system is warranted.

**Supplementary Information:**

The online version contains supplementary material available at 10.1186/s12889-025-22215-3.

## Introduction

Population aging is a global phenomenon that poses multiple challenges to both the health and economic sustainability of societies. It is characterized not only by increasing healthcare and pension expenditures but also by shrinking workforces. According to the report by the World Bank [1], the East Asian and Pacific region is experiencing a particularly rapid aging process compared to other parts of the world. In Taiwan, for instance, the proportion of the population aged over 65 exceeded 14% in 2018 and is projected to exceed 20% by 2025. In response to these demographic and societal challenges, many European countries have implemented "extending working lives" policies since the 1990s [2–4]. In Taiwan, however, pension systems are less comprehensive, and policy reforms to address population aging have been implemented much later. The National Pension Insurance was established in 2008 to provide basic coverage for individuals not included in occupation-based pension schemes. In 2019, the Middle-aged and Elderly Employment Promotion Act was enacted to promote labor force participation among middle-aged and older individuals. Given Taiwan’s later but faster population aging, extending working lives has become an urgent policy challenge requiring innovative solutions beyond simply adopting European models.

The traditional concept of retirement is facing challenges due to the rise in nonstandard/temporary employment, and the rapid evolution of technology and required skills in the workplace. Raising the statutory retirement age has been one of the most common policies designed to foster longer working lives. Policies aimed at extending working lives need to go beyond merely postponing retirement ages [5]. In the context of improving work conditions, systematic reviews revealed that high physical demands, shift work, and low job control were associated with early retirement [6, 7]. However, there is high heterogeneity in the occupation backgrounds of study participants and variations in the measurements for work conditions.

Moreover, most existing studies have been conducted in Europe, where pension systems are generally more generous and comprehensive [8]. Only a few of studies have been conducted in countries with less developed pension systems. For instance, a study in China found that poor health is associated with labor market exit, particularly among farmers [9]. In Taiwan, pension systems are primarily occupation-based, supplemented by the National Pension Insurance. Generally, old-age pensions are not tied to recipients’ financial conditions, except for means-tested social assistance benefits. The design of pension systems can significantly influence work and retirement decisions and may contribute to income and health inequalities in old age [10]. In such case, extending working lives has raised concerns about forcing employees with poor health and low income to continue working to secure retirement benefits [5]. Some Taiwanese workers, particularly those in unstable jobs, small enterprises, or self-employment, do not receive pension benefits. These workers often endure adverse working conditions and a heightened risk of work-related illnesses and disabilities, which can impede their ability to work until old age. In contrast, employees with secure pension benefits tend to have stable jobs, better-regulated working conditions, lower health risks, and higher motivation to work until old age to become eligible for pension benefit upon retirement. However, to our knowledge, no research has examined how pension availability affects the labor market participation of older workers.

Health issues, whether physical or mental, emerge as a predominant obstacle to employment among older adults [11–14]. Higher level of work-related stress not only increased the likelihood of early labor market exit but also poor health [15]. Numerous studies have demonstrated the correlation between work conditions (e.g., high psychological job demands, low autonomy, limited influence, insufficient social support) and poor physical health in middle-aged and older adults [16, 17]. Notably, the significant effect of work-related stress on labor market exit disappears when accounting for workers' health status [18]. This suggests that health partially mediates the relationship between psychosocial work conditions and early labor market exit [19]. Nevertheless, the direct measurement of reasons for labor market exit remains limited, and obtaining such information would contribute to a more comprehensive knowledge that can inform the development of policies aimed at modifying work environment and thereby preventing labor market exit for health reasons among older employees.

While health issues are expected to influence labor market performance of older workers, few studies have examined the relationship between work conditions and labor market exit for health reasons. Additionally, there is a scarcity of studies available regarding the association between work conditions and labor market exit in East Asian countries, where population aging is occurring at a faster pace than elsewhere and pension system are less comprehensive. The study aim was to examine the influences of work conditions on labor market exit for health reasons, as opposed to old age retirement, among middle-aged and older adults with different pension statuses.

## Methods

### Study design

We employed data from the Healthy Aging Longitudinal Study in Taiwan (HALST), a nationally representative longitudinal cohort of older adults, and the Occupational Safety and Health Surveys (OSHS). This OSHS dataset was utilized to derive occupational-level work conditions for linkage with the HALST data. All data were de-identified before being handled to the authors.

The HALST study employed a systematic sampling method to enroll healthy community-dwellers aged 55 and above from seven selected districts in Taiwan. These seven locations encompassed both urban and rural areas, as well as to represent various ethnic groups, thereby reflecting the diverse sociodemographic characteristics of the Taiwanese population [20]. The initial wave of the survey was conducted from 2009 to 2013, followed by a second wave of data collection from 2014 to 2019. A total of 5,663 participants completed the first wave of the survey, while 4,233 participants completed the second wave. In this study, data from both waves of the survey were utilized.

The OSHS, conducted by the Ministry of Labor of Taiwan, has been administered to a nationwide working population every 3–5 years since 1988, with the goal of gaining insights into workplace safety and health conditions. For each survey of the OSHS, participants were selected using a two-stage random sampling process. In the first stage, all districts and villages throughout Taiwan were grouped into strata based on their levels of urbanization, and a random sample of districts and villages was selected from each stratum. In the second stage, households were then randomly selected within each district or village. Individuals who were currently employed at the time of the survey were identified from the sampled households. Participation was limited to individuals who were economically active, and those who were not economically active were deemed ineligible. The interviews were conducted face-to-face by trained interviewers. The sample's representativeness was carefully ensured, and detailed information regarding the survey's sampling methodology can be found elsewhere [21].

### Measurement

#### Employment and reasons for labor market exit

**Fig. 1:**
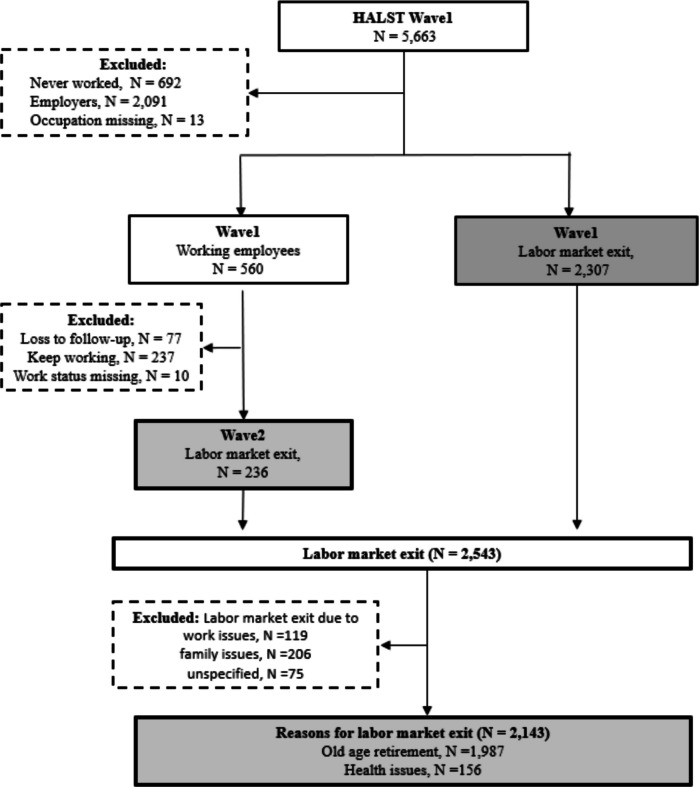
Flow chart for study sample selection from the Healthy Aging Longitudinal Study in Taiwan cohort

In HALST, participants were asked about their work history and whether they were currently working at the time of the survey. Individuals who had never worked or were employers were excluded from wave 1 survey (*N* = 692 and 2091, Fig. 1). Those who had been employees self-reported their last primary occupation, defined as “the job you have worked at the longest or the main source of your income.” A total of 2307 participants in wave 1 and another 236 participants in wave 2 reported having worked previously but were not currently employed (Fig. 1). They were then asked to select the reason for their labor market exit from: old age retirement, health reasons, work-related issues (being laid off, difficulty adapting to work, or unsatisfactory pay), family-related reasons (marriage or caregiving burden), and other unspecified factors. To examine the work conditions as risk factors for labor market exit for health reasons compared to old age retirement, further exclusions were made for those who exited the labor market for reasons other than old age retirement or health reasons (*n* = 400), resulting in 2,143 participants for the analysis. If both old-age retirement and health reasons were selected (*n* = 25), participants were categorized into the old-age retirement group.

#### Work conditions

In the OSHS in 2007, the occupations were categorized according to the 5th edition of the Standard Occupational Classification by the Ministry of Labor of Taiwan, which aligns with the International Standard Classification of Occupations (ISCO-88) using two-digit major and sub-major groups. A job exposure matrix was created by aggregating work conditions for 35 occupations using data from 3,148 individuals [22], by averaging scores for each work condition among employees aged 50 and above (supplementary Table 1). The response rates for this survey wave was 86%. Participants provided information regarding their total working hours for the week preceding the survey. The psychosocial work conditions measured in OSHS surveys were psychological job demands, job control, workplace justice, and physical demands. Psychological job demands and job control were assessed using the validated Chinese version of the Job Content Questionnaire (JCQ) based on the job-demand-control (JDC) model developed by Karasek and Theorell [23, 24]. The OSHS questionnaires in 2007 included nine items for the job control scale (learning new things, non-repetitive work, creative work, allowing own decision, high level of skills, freedom to make decisions, various tasks, influential opinions, and the development of one’s abilities), as well as seven items for psychological job demands (fast work, hard work, excessive work, insufficient time, concentration on the job for a long time, hectic work, and insufficient manpower). The items were presented as statement, and respondents were asked to indicate their level of agreement on a four-point Likert scale ranging from 1 (strongly disagree) to 4 (strongly agree).

A seven-item scale for the assessment of workplace justice (trust, reliable information, fair work arrangements, fair rewards, fair performance evaluation, inclusion of information in decision-making, and respect) was utilized. This scale was modified from the original standard questionnaire and showed good psychometric properties [25, 26]. Responses for these items were recorded on a four-point Likert scale, ranging from one (strongly disagree) to four (strongly agree).

One item assessing physical demands (my job requires a lot of physical effort) was evaluated using a four-point Likert scale, with responses ranging from one (strongly disagree) to four (strongly agree). The scores for job control, psychological demands, workplace justice, and physical demands were summed, standardized, and subsequently divided into low and high groups based on the median.

#### Socioeconomic and health variables

In HALST, pension availability was evaluated by asking participants about their primary financial sources in the previous year. Participants were required to select only one option from the following: retirement pensions or National Pension, support from children or family members, rental or stock dividends, and social welfare benefits or others. Individuals who obtained retirement pensions or National Pension were classified as having a pension, regardless of other financial resources. Those who did not receive either type of pension were categorized as having no pension.

Participants self-reported their gender, birth year, educational years, marital status, household income, and pension availability. Education level was categorized into two groups: primary education or less, and secondary education or above. Marital status was grouped into married and other status (separated, divorced, widowed, or single). Participants provided information about their household income by selecting one of seven predefined categories. However, 42.2% of participants either left this information as unknown or chose not to disclose it. To address this, a dummy variable was generated to account for the missing household income data. Additionally, participants were categorized into two groups—high and low household income—using a threshold of 30,000 New Taiwan Dollars per month (equivalent to approximately 945 U.S. Dollars).

Mental health and physical health were both assessed through questionnaires. Depressive symptoms were assessed using the 20-item Center for Epidemiological Studies-Depression Scale (CES-D) [27]. Response options for each item ranged from 0 to 3. Scores on the scale could range from 0 to 60, with higher scores indicating a higher level of depressive symptoms. Physical illnesses were categorized based on whether participants reported a history of two or more of the major diseases: hypertension, diabetes, cardiovascular disease, stroke, or cancer.

### Statistical analysis

We conducted Chi-square tests and Mann–Whitney tests to compare the differences between old age retirement and labor market exit for health reasons. Because HALST participants were nested within occupations, we employed a mixed regression analysis with fixed effects for work conditions on labor market exit for health reasons. This model included work conditions that have been reported to be associated with health, namely job control, psychological job demands, physical demands, workplace justice, and weekly working hours. The odds ratio (OR) and 95% confidence interval (CI) were estimated, and adjusted models were utilized, controlling for gender, age, education, marital status, household income, and pension availability. To investigate how pension availability influenced the impact of work conditions on labor market exit, we further stratified the participants by their pension availability and examined the associations between work conditions and labor market exit for health reasons separately. Data analyses were performed using SAS 9.4 (SAS Institute Inc., Cary, NC, USA).

## Results

Among the 2,143 study participants, 156 (7.3%) reported labor market exit for health reasons. Compared to old age retirement, participants who exited the labor market for health reasons were generally younger, had a higher proportion of female, and a lower level of education, being unmarried, had lower household income, and lacked pension coverage (Table 1). Additionally, they reported higher depressive symptom scores and a higher prevalence of having physical illness when compared to those who retired due to old age. Moreover, participants who exited the labor market for health reasons were more likely to have jobs characterized by low workplace justice, low job control, and higher physical demands. Notably, 60.15% of all participants reported having a pension.

**Table 1:** Characteristics of individuals who exited labor market for health reasons or old age retirement

	All	Health reasons	Old age retirement	*p*
	(*n* = 2143)	(*n* = 156)	(*n* = 1987)	
	N (%)	N (%)	N (%)	
**Demographic characteristics**				
Gender				< 0.001
Female	849 (39.62)	92 (58.97)	757 (38.10)	
Male	1294 (60.38)	64 (41.03)	1230 (61.90)	
Age (year)				< 0.001
< 65	579 (27.02)	70 (44.87)	509 (25.62)	
≥ 65	1564 (72.98)	86 (55.13)	1478 (74.38)	
Education				< 0.001
Primary or lower	802 (37.44)	106 (67.95)	696 (35.05)	
Secondary or above	1340 (62.56)	50 (32.05)	1290 (64.95)	
Marital status				< 0.001
Married	1624 (75.78)	93 (59.62)	1531 (77.05)	
Other status	519 (24.22)	63 (40.38)	456 (22.95)	
**Socioeconomic status**				
Household income				0.002
High	643 (30.00)	33 (21.15)	610 (30.70)	
Low	596 (27.81)	61 (39.10)	535 (26.93)	
Missing	904 (42.18)	62 (39.74)	842 (42.38)	
Pension				< 0.001
No	854 (39.85)	88 (56.41)	766 (38.55)	
Yes	1289 (60.15)	68 (43.59)	1221 (61.45)	
**Health conditions**				
CES-D scores (mean and SD)	4.05 (6.22)	8.83 (9.67)	3.68 (5.70)	< 0.001
Physical illness				0.004
Yes	592 (27.62)	59 (37.82)	533 (26.82)	
No	1551 (72.38)	97 (62.18)	1454 (73.18)	
**Work conditions**				
Psychological job demands				0.158
High	1085 (50.63)	70 (44.87)	1015 (51.08)	
Low	1058 (49.37)	86 (55.13)	972 (48.92)	
Job control				< 0.001
High	1041 (48.58)	27 (17.31)	1014 (51.03)	
Low	1102 (51.42)	129 (82.69)	973 (48.97)	
Physical demands				< 0.001
High	1064 (49.65)	130 (83.33)	934 (47.01)	
Low	1079 (50.35)	26 (16.67)	1053 (52.99)	
Workplace justice				< 0.001
High	1063 (49.60)	52 (33.33)	1011 (50.88)	
Low	1080 (50.40)	104 (66.67)	976 (49.12)	
Working hours (mean and SD)	42.25 (3.59)	42.38 (3.39)	42.24 (3.60)	0.990

In the adjusted regression model (Table 2), study participants who had experienced low job control (adjusted OR = 2.23, 95% CI = 1.05 to 4.73, *p* = 0.037) and high physical demands (adjusted OR = 2.72, 95% CI = 1.26 to 5.85, *p* = 0.012) were more likely to exit the labor market for health reasons rather than due to old age retirement. Having no pension was associated with labor market exit for health reasons as well (adjusted OR = 1.74, 95% CI = 1.19 to 2.54, *p* = 0.005).

**Table 2:** The association of working conditions and pension availability with labor market exit for health reasons

	Crude model		Adjusted model	*p*
Independent variables	OR (95%CI)		OR (95%CI)	
Psychological Job demands		0.773		0.398
Low	1		1	
High	0.89 (0.41–1.97)		0.80 (0.47–1.36)	
Job control		< 0.001		0.037
High	1		1	
Low	5.07 (2.64–9.72)		2.23 (1.05–4.73)	
Physical demands		< 0.001		0.012
Low	1		1	
High	4.98 (2.70–9.20)		2.72 (1.26–5.85)	
Workplace justice		0.071		0.534
High	1		1	
Low	2.04 (0.94–4.43)		0.82 (0.44–1.55)	
Working hours	0.98 (0.87–1.10)	0.741	0.95 (0.87–1.03)	0.194
Pension		0.001		0.005
Yes	1		1	
No	1.97 (1.37–2.84)		1.74 (1.19–2.54)	

The model’s outcome was labor market exit for health reasons, as opposed to old age retirement. Odds ratios (OR) and 95% confidence intervals (CI) were shown in univariate crude models and mutually adjusted models, with additional adjustments for gender, age, education, marital status, and household income

Compared to those who had pension, participants without pension had higher proportion of low job control, high physical demands, and low workplace justice (Supplementary Table 2). We further stratified the participants by their pension status in the logistic regression analysis (Table 3). Among older adults who had no pension coverage, those who had experienced low job control (OR = 4.58, 95% CI = 1.80 to 11.71, *p* = 0.002) and high physical demands (OR = 3.03, 95% CI = 1.28 to 7.17, *p* = 0.013) were more likely to exit the labor market for health reasons rather than due to old age retirement, but psychological job demands showed a negative association (OR = 0.52, 95% CI = 0.30 to 0.90, *p* = 0.020). Among those with pension, work conditions were not significantly associated with labor market exit for health reasons.

**Table 3:** Association between working conditions and labor market exit for health reasons, stratified by pension availability

		No pension (*N* = 854)		Pension (*N* = 1289)	
Work conditions		OR (95%CI)	*p*	OR (95%CI)	*p*
Psychological Job demands			0.020		0.693
Low		1		1	
High		0.52 (0.30–0.90)		1.17 (0.53–2.60)	
Job control			0.002		0.783
High		1		1	
Low		4.58 (1.80–11.71)		0.87 (0.30–2.49)	
Physical demands			0.013		0.117
Low		1		1	
High		3.03 (1.28–7.17)		2.43 (0.79–7.47)	
Workplace justice			0.052		0.316
High		1		1	
Low		0.51 (0.26–1.01)		1.61 (0.62–4.18)	
Working hours		0.93 (0.84–1.02)	0.117	0.96 (0.85–1.09)	0.543

The model’s outcome was labor market exit for health reasons, as opposed to old age retirement. Models were mutually adjusted for gender, age, education, marital status, and household income

Given that participants who exited the labor market for health reasons were younger than the age of those who reported old age retirement, we stratified the participants by age (< 65 and ≥ 65 years old) and examined the association in each group separately (Table 4). High physical demands were associated with labor market exit for health reasons in the older group (OR = 3.21, 95% CI = 1.15 to 8.93, *p* = 0.027), while low job control was associated with labor market exit for health reasons in the younger group (OR = 3.68, 95% CI = 1.28 to 10.63, *p* = 0.018).

**Table 4:** Association between working conditions and labor market exit for health reasons, stratified by age

		Age < 65 (*N* = 579)		Age ≥ 65 (*N* = 1564)	
Work conditions		OR (95%CI)	*p*	OR (95%CI)	*p*
Psychological Job demands			0.345		0.551
Low		1		1	
High		0.69 (0.32–1.51)		0.83 (0.44–1.57)	
Job control			0.018		0.464
High		1		1	
Low		3.68 (1.28–10.63)		1.43 (0.54–3.78)	
Physical demands			0.079		0.027
Low		1		1	
High		2.58 (0.89–7.47)		3.21 (1.15–8.93)	
Workplace justice			0.410		0.500
High		1		1	
Low		0.69 (0.27–1.73)		0.77 (0.36–1.68)	
Working hours		0.88 (0.78–1.00)	0.050	0.99 (0.89–1.09)	0.765

The model’s outcome was labor market exit for health reasons, as opposed to old age retirement. Models were mutually adjusted for gender, education, marital status, and household income

## Discussion

Compared to old age retirement, low job control and high physical demands were risk factors for labor market exit for health reasons. Approximately 40% of older adults did not have any pension at the time of labor market exit, and no pension coverage was associated with labor market exit for health reasons. Additionally, work conditions were found to be associated with labor market exit for health reasons specifically among older adults who had no pension, but not those who were receiving pension benefits.

As expected, this study reveals a correlation between high physical demands and labor market exit for health reasons, particularly among older workers. As workers age, physical demands increasingly become a barrier to extended working lives. Previous studies also demonstrated that senior workers engaged in primarily physical demanding jobs tend to exhibit higher stress scores [28]. Furthermore, research indicates that a favorable change in physical workload lowered the risk of exiting paid employment [29]. Beyond physical demands, psychosocial work conditions play a crucial role in maintaining older workers in the workplace. Even for senior workers engaged in physically demanding jobs, psychosocial work stress has been associated with increased perceived stress and early retirement [30–33]. According to the JDC model, the combination of low job control and low job demands characterizes passive work, which is associated with adverse mental and physical health [34, 35]. This finding echoes our observation that low job control and low psychological demands were associated with labor market exit for health reasons. With aging, the importance of job control, including skill use and decision authority, in predicting prolonged working lives increased [36]. This study found that low job control significantly impacted participants younger than 65 years old, a group that often occupies high occupational positions and typically experiences high job control. Previous research has demonstrated an association of low job control with poor physical and mental health outcomes [37, 38], which may consequently impede an individual's ability to work until traditional retirement age. Furthermore, decision authority, a component of job control, buffers the negative impact of heavy physical demands on working longer among older men [39, 40]. Therefore, to retain older workers in the labor market, implementing work redesign strategies focused on enhancing skill utilization and fostering an environment that promotes decision authority may be beneficial.

While high psychological job demands are commonly perceived as work stress, this study found an association with a lower risk of labor market exit for health reasons among individuals without pension support. Similarly, a previous study also noted that a decrease in psychological job demands increased the likelihood of exiting paid employment [29]. It has been proposed that when an older worker experiences physical functional limitation, psychological job demands may become the last resource to prevent exiting from paid employment [29]. High psychological demands, a component of active jobs within the JDC model, may protect workers from dementia through cognitive stimulation [34], thereby reducing the risk of labor market exit for health reasons. Furthermore, psychological job demands were generally more pronounced for workers with higher occupational positions and educational levels [41–45]. In this study, participants without pensions were younger, had lower levels of education, and reported higher physical demands compared to those with pensions. Among participants without pensions, jobs with low psychological demands often involved high physical demands, potentially increasing the risk of labor market exit due to health reasons.

In this study, having no pension was associated with labor market exit for health reasons, likely because pension eligibility is tied to old age retirement. Additionally, adverse work conditions were associated with labor market exit for health reasons in the no-pension group. A cumulative social disadvantage in their work life was evident in individuals without pension coverage who also experienced poor health. In other words, individuals experiencing greater marginalization in the labor market are less likely to reap benefits from the pension system in their later years. Previous studies have revealed a similar predicament among low-educated workers, who experienced more lost working years due to unemployment, lack of income, and disability benefits [44, 46]. As the occurrence of chronic diseases was highest among low educated workers, the probabilities of exiting paid employment through disability benefits were high among this group [47]. The associations between physical demands, autonomy, and job strain and health were also strongest in the lower educated workers [48]. The findings of this study suggest an accumulation of disadvantages of adverse work conditions, poor health, and financial insecurity in old age.

The strength of this study is that we used data from a nationally representative cohort of middle-aged and older adults. Using self-reported reasons for labor market exit has both pros and cons. It is subject to recall bias, especially among those who retired a substantial time before the survey. Nevertheless, we captured employees who were not accessible in registered database of labor statistics, such as informal employment and irregular employment. This is evidenced by the high proportion of participants receiving no pension, and the well-being of marginalized employees deserves more attention. This study has limitations. First, because work conditions were not collected in the HALST cohort, they were aggregated by occupation from another national survey (the OSHS). There could be variations across employees in the same occupation, which introduce bias. Extreme work conditions may have been eliminated during the aggregation process, thus limiting the variations. Second, work stress was evaluated using the JDC model in this study, omitting other useful work stress models. For example, health mediated the association between work stress, measured by effort-reward imbalance, and early labor market exit [18]. Including more work stress measurements in this study would provide a more comprehensive picture of the association between work conditions and labor market exit. Third, the cross-sectional design of this study poses limitations on information including the precise timing of labor market exits and exposure to work conditions, as well as whether the exits were temporary or permanent.

In conclusion, to mitigate early labor market exit for health reasons and to encourage the retention of workers in their later years, organizational interventions and labor policies should strive to promote active job design while reducing physical demands. This study also underscores the necessity for targeted policies for individuals lacking secure pension coverage, such as older adults with fragmented work tenure and non-standard employment, to support for continued employment and to establish an inclusive pension system.

## Supplementary Information

Supplementary Material 1

## Data Availability

The data that support the findings of this study are available from the National Health Research Institutes of Taiwan. The availability of these data is subject to restrictions, and their use in this study was permitted under license.

## Abbreviations

CES-D: Center for Epidemiological Studies-Depression Scale
CI: Confidence Interval
HALST: Healthy Aging Longitudinal Study in Taiwan
ISCO: International Standard Classification of Occupations
JCQ: Job Content Questionnaire
OR: Odds Ratio
OSHS: Occupational Safety and Health Surveys
