# Supplementary material for: Influence of pension availability on the association between work conditions and labor market exit for health reasons: evidence from a Taiwanese older adults cohort
Source: BMC Public Health. 2025 Mar 17;25:1022. doi: 10.1186/s12889-025-22215-3 (PMC11912721; doi:10.1186/s12889-025-22215-3)
Supplement: Supplementary file 1 — Supplementary Material 1 [file 12889_2025_22215_MOESM1_ESM.docx]

Supplementary table 1. Work conditions of occupations derived from occupational safety and health survey (OSHS) in 2007.

| Occupation sub-major groups | N (%) | Psychological job demands | Job control | Physical demands | Workplace justice | Working hours |
| --- | --- | --- | --- | --- | --- | --- |
|  |  | Mean (SD) | Mean (SD) | Mean (SD) | Mean (SD) | Mean (SD) |
| Legislators and government administrators | 47 (1.49) | 47.52 (14.27) | 64.66 (8.97) | 40.43 (20.78) | 66.87 (14.59) | 45.72 (7.77) |
| Directors and chief executives | 23 (0.73) | 47.62 (13.00) | 68.36 (10.28) | 36.23 (22.28) | 70.19 (13.18) | 42.78 (11.51) |
| Production and operations managers | 58 (1.84) | 51.97 (11.91) | 64.37 (13.83) | 39.08 (18.87) | 61.82 (13.98) | 44.93 (6.76) |
| Other managers | 25 (0.79) | 49.9 (8.38) | 65.11 (11.57) | 37.33 (11.06) | 61.71 (13.71) | 43.16 (3.84) |
| Physical, mathematical and engineering science professionals | 45 (1.43) | 53.97 (11.40) | 65.25 (8.55) | 37.78 (15.24) | 57.57 (14.39) | 42.31 (7.29) |
| Biological science and health professionals | 32 (1.02) | 50.30 (13.25) | 61.11 (14.46) | 42.71 (21.14) | 58.33 (14.36) | 39.41 (12.49) |
| Teachers | 115 (3.65) | 50.23 (12.65) | 66.91 (13.29) | 41.16 (20.86) | 61.37 (14.83) | 35.99 (13.81) |
| Accountants and business professionals | 4 (0.13) | 55.95 (22.12) | 72.22 (20.41) | 25.00 (16.67) | 72.62 (18.80) | 42.00 (4.00) |
| Lawyers and legal professionals | 2 (0.06) | 59.52 (16.84) | 68.06 (9.82) | 33.33 (0.00) | 71.43 (6.73) | 45.00 (7.07) |
| Social science and related professionals | 1 (0.03) | 66.67 (-) | 36.11 (-) | 33.33 (-) | 52.38 (-) | 40.00 (-) |
| Other professionals | 20 (0.64) | 48.33 (11.17) | 56.39 (13.49) | 38.33 (16.31) | 63.33 (6.75) | 47.20 (8.98) |
| Physical and engineering science associate professionals | 115 (3.65) | 52.92 (10.15) | 56.30 (11.89) | 51.88 (19.84) | 57.06 (15.08) | 42.34 (6.17) |
| Biological science and health associate professionals | 11 (0.35) | 47.19 (11.56) | 56.31 (13.44) | 45.45 (16.82) | 56.71 (12.5) | 40.73 (8.55) |
| Teaching and related associate professionals | 8 (0.25) | 45.83 (16.29) | 68.40 (18.36) | 45.83 (24.80) | 58.33 (15.43) | 29.13 (14.30) |
| Finance and business services associate professionals | 113 (3.59) | 49.22 (11.05) | 58.21 (9.93) | 43.66 (19.96) | 62.75 (12.71) | 44.96 (7.45) |
| Government administrative officials and business clerical supervisors | 144 (4.57) | 52.65 (13.61) | 59.80 (10.30) | 42.13 (21.59) | 60.85 (15.95) | 42.62 (5.78) |
| Administrative associate professionals | 106 (3.37) | 47.80 (13.06) | 53.80 (12.57) | 37.42 (18.80) | 60.96 (16.93) | 41.07 (3.80) |
| Customs, tax and government associate professionals | 4 (0.13) | 51.19 (17.98) | 52.78 (6.00) | 41.67 (16.67) | 64.29 (2.75) | 38.00 (4.00) |
| Other associate professionals | 14 (0.44) | 50.68 (12.89) | 60.12 (7.75) | 47.62 (17.12) | 63.95 (12.21) | 44.57 (14.85) |
| Office clerks | 217 (6.89) | 48.39 (11.18) | 50.27 (12.62) | 42.40 (19.11) | 58.90 (13.63) | 41.23 (5.87) |
| Personal services workers | 173 (5.5) | 49.77 (10.57) | 48.59 (14.04) | 53.37 (21.79) | 60.56 (13.97) | 47.46 (13.26) |
| Protective services workers | 90 (2.86) | 44.97 (9.00) | 45.86 (12.17) | 42.96 (20.14) | 57.20 (13.27) | 50.78 (11.66) |
| Models, salespersons and demonstrators | 58 (1.84) | 47.54 (10.87) | 48.28 (13.13) | 47.13 (20.74) | 60.59 (14.51) | 46.28 (10.55) |
| Agricultural, animal husbandry, forestry and fishing workers | 82 (2.60) | 49.59 (9.52) | 42.48 (12.80) | 61.38 (17.74) | 56.79 (15.96) | 41.21 (11.40) |
| Extraction and building trades workers | 304 (9.66) | 53.84 (10.25) | 48.89 (11.94) | 69.41 (18.26) | 57.55 (14.48) | 39.04 (10.12) |
| Metal and machinery workers | 136 (4.32) | 52.80 (10.66) | 52.14 (14.71) | 61.27 (21.17) | 59.31 (14.93) | 42.33 (8.36) |
| Precision, handicraft and printing workers | 7 (0.22) | 54.42 (9.47) | 39.68 (5.49) | 47.62 (26.23) | 63.27 (12.81) | 43.00 (6.30) |
| Other craft and related trades workers | 26 (0.83) | 50.73 (12.70) | 46.05 (10.92) | 57.69 (20.13) | 67.03 (11.81) | 45.42 (8.70) |
| Stationary-plant and related operators | 60 (1.91) | 51.03 (10.88) | 47.50 (11.29) | 55.56 (20.96) | 58.02 (11.28) | 43.50 (7.33) |
| Machine operators | 367 (11.66) | 51.65 (11.14) | 46.00 (13.26) | 53.95 (21.66) | 56.68 (14.92) | 44.30 (6.99) |
| Assemblers | 51 (1.62) | 50.14 (12.68) | 43.9 (13.02) | 47.71 (26.04) | 57.52 (13.33) | 40.29 (9.63) |
| Drivers and mobile-plant operators | 162 (5.15) | 50.18 (9.79) | 47.53 (11.39) | 56.79 (22.26) | 56.38 (14.01) | 44.75 (11.43) |
| Sales and services elementary occupations | 347 (11.02) | 49.6 (12.13) | 42.11 (13.85) | 59.08 (23.16) | 58.82 (15.61) | 43.25 (9.77) |
| Production laborers and related workers | 100 (3.18) | 50.24 (11.00) | 38.39 (13.20) | 62 (22.73) | 54.76 (14.56) | 39.64 (8.88) |
| Other elementary occupations | 20 (0.64) | 41.9 (6.66) | 37.64 (14.06) | 46.67 (25.13) | 52.38 (18.08) | 35.75 (7.34) |

Supplementary Table 2. Characteristics of individuals with and without pension availability.

|  | No Pension |  | Pension | *p* |
| --- | --- | --- | --- | --- |
|  | (N = 854) |  | (N = 1289) |  |
|  | N (%) |  | N (%) |  |
| **Sociodemographic characteristics** | |  |  |  |
| Gender |  |  |  | 0.171 |
| Female | 354 (41.45) |  | 495 (38.40) |  |
| Male | 500 (58.55) |  | 794 (61.60) |  |
| Age (year) |  |  |  | <0.001 |
| <65 | 274 (32.08) |  | 305 (23.66) |  |
| ≥65 | 580 (67.92) |  | 984 (76.34) |  |
| Education |  |  |  | <0.001 |
| Primary or lower | 360 (42.15) |  | 442 (34.32) |  |
| Secondary or above | 494 (57.85) |  | 846 (65.68) |  |
| Marital status |  |  |  | 0.732 |
| Married | 651 (76.23) |  | 973 (75.48) |  |
| Other status | 203 (23.77) |  | 316 (24.52) |  |
| Household income |  |  |  | <0.001 |
| High | 201 (23.54) |  | 442 (34.29) |  |
| Low | 224 (26.23) |  | 372 (28.86) |  |
| Missing | 429 (50.23) |  | 475 (36.85) |  |
| **Health conditions** |  |  |  |  |
| CES-D scores (mean and SD) | 4.28 (6.68) |  | 3.90 (5.89) | 0.227 |
| Physical illness |  |  |  | 1.000 |
| Yes | 236 (27.63) |  | 356 (27.62) |  |
| No | 618 (72.37) |  | 933 (72.38) |  |
| **Work conditions** |  |  |  |  |
| Psychological Job demands |  |  |  | 0.182 |
| High | 448 (52.46) |  | 637 (49.42) |  |
| Low | 406 (47.54) |  | 652 (50.58) |  |
| Job control |  |  |  | 0.001 |
| High | 377 (44.15) |  | 664 (51.51) |  |
| Low | 477 (55.85) |  | 625 (48.49) |  |
| Physical demands |  |  |  | <0.001 |
| High | 496 (58.08) |  | 568 (44.07) |  |
| Low | 358 (41.92) |  | 721 (55.93) |  |
| Workplace justice |  |  |  | 0.017 |
| High | 396 (46.37) |  | 667 (51.75) |  |
| Low | 458 (53.63) |  | 622 (48.25) |  |
| Working hours (mean and SD) | 42.61 (3.22) |  | 42.01 (3.79) | <0.001 |

*Abbreviations*: CES-D = Center for Epidemiological Studies-Depression Scale.

Notes: Differences between groups were examined by chi-square tests and Mann-Whitney tests
